# Efficiently activated ε‐poly‐L‐lysine production by multiple antibiotic‐resistance mutations and acidic pH shock optimization in *Streptomyces albulus*

**DOI:** 10.1002/mbo3.728

**Published:** 2018-10-08

**Authors:** Liang Wang, Shu Li, Junjie Zhao, Yongjuan Liu, Xusheng Chen, Lei Tang, Zhonggui Mao

**Affiliations:** ^1^ The Key Laboratory of Industrial Biotechnology Ministry of Education School of Biotechnology Jiangnan University Wuxi Jiangsu China; ^2^ College of Marine Science Shandong University (Weihai) Weihai China

**Keywords:** ε‐PL overproduction, acid pH shock optimization, high production analysis, multiple antibiotic‐resistance mutations, ribosome engineering, *Streptomyces*

## Abstract

ε‐Poly‐L‐lysine (ε‐PL) is a food additive produced by *Streptomyces* and is widely used in many countries. Working with *Streptomyces albulus* FEEL‐1, we established a method to activate ε‐PL synthesis by successive introduction of multiple antibiotic‐resistance mutations. Sextuple mutant R6 was finally developed by screening for resistance to six antibiotics and produced 4.41 g/L of ε‐PL in a shake flask, which is 2.75‐fold higher than the level produced by the parent strain. In a previous study, we constructed a double‐resistance mutant, SG‐31, with high ε‐PL production of 3.83 g/L and 59.50 g/L in a shake flask and 5‐L bioreactor, respectively. However, we found that R6 did not show obvious advantages in fed‐batch fermentation when compared with SG‐31. For further activation of ε‐PL synthesis ability, we optimized the fermentation process by using an effective acidic pH shock strategy, by which R6 synthetized 70.3 g/L of ε‐PL, 2.79‐fold and 1.18‐fold greater than that synthetized by FEEL‐1 and SG‐31, respectively. To the best of our knowledge, this is the highest reported ε‐PL production to date. This ε‐PL overproduction may be due to the result of R99P and Q856H mutations in ribosomal protein S12 and RNA polymerase, respectively, which may be responsible for the increased transcription of the ε‐poly‐lysine synthetase gene (*pls*) and key enzyme activities in the Lys synthesis metabolic pathway. Consequently, ε‐PL synthetase activity, intracellular ATP, and Lys concentrations were improved and directly contributed to ε‐PL overproduction. This study combined ribosome engineering, high‐throughput screening, and targeted strategy optimization to accelerate ε‐PL production and probe the fermentation characteristics of hyperyield mutants. The information presented here may be useful for other natural products produced by *Streptomyces*.

## 1 INTRODUCTION

ε‐Poly‐L‐Lysine (ε‐PL) is a natural homopolymer of microbial origin, where 25–35 L‐lysine monomers are linked by peptide bonds between α‐carboxyl and ε‐amino groups. Currently, ε‐PL is in increasing demand because of its clinical effects and biological and chemical activities (Shima, Matsuoka, Iwamoto, & Sakai, 1984). As it is soluble, biodegradable, edible, and nontoxic toward humans and the environment, ε‐PL and its derivatives have been widely used as a food preservative and as biodegradable fibers, drug carriers, dietary agents, emulsifying agents, and highly water absorbable hydrogels in many countries, such as Japan, Korea, the United States, and China (Shih, Shen, & Van, 2006). The industrialization prospect of ε‐PL is considered to be very promising. In 1947, Eprain et al. tried to chemically synthesize poly‐lysine, but the product was toxic and could not be used as a biological preservative (Shima & Sakai, 1981). In 1977, the first ε‐PL‐producing strain, *S. albulus* 346, was identified (Shima & Sakai, 1977). Since ε‐PL can only be synthesized by microorganisms, screening and breeding hyper‐ε‐PL‐producing strains is a better choice to obtain ε‐PL to meet the needs of industrial production.

Over the past 40 years, many soil microorganisms have been identified in the search for the ideal ε‐PL producers, including *S. albulus*,* S. lydicus*,* Kitasatospora* sp., and *S. griseofuscus* (Li et al., 2011; Shima & Sakai, 1981; Xia, Xu, Feng, Xu, & Chi, 2013). Among them, *S. albulus* is considered to be the best ε‐PL producer as it can produce more ε‐PL than others (Xu et al., 2016). Since then, many efforts have been made for enhancing ε‐PL productivity, but more attention has been paid to medium composition and fermentation process optimization (Chen et al., 2011; Geng et al., 2014; Xia, Xu, Xu, Feng, & Bo, 2014). However, further increases in ε‐PL production have been difficult to achieve by conventional methods alone.

Recently, efforts have been made to increase ε‐PL production using molecular biology methods. The Vitreoscilla hemoglobin (*VHb*) gene was integrated into the chromosome of *S. albulus* PD‐1, and ε‐PL biosynthesis was enhanced from 22.7 to 34.2 g/L in fed‐batch fermentation (Xu et al., 2015). The heterologous *VHb* gene and SAM synthetase gene (*metK*) were also overexpressed in the *S. albulus* NK660 chromosome, resulting in an increased biomass (1.14‐fold) and ε‐PL production (1.27‐fold) (Gu et al., 2016). However, there is still room for strain improvement. This result may have occurred as ε‐PL biosynthesis is tightly controlled and highly regulated, and modifications to only one or two genes do not result in an increase in ε‐PL production. Moreover, it is possible that the lack of some promoters can also restrict an increase in ε‐PL production (Wang, Gao, Tang, Hu, & Wu, 2015).

Classical methods, such as physical and chemical mutagenesis, are still effective without applying genomic information or genetic tools to develop highly productive strains. In 1998, a successful trial involved the use of S‐(2‐aminoethyl)‐L‐cysteine (AEC, an L‐lysine analog) as the selective marker for the enhancement of ε‐PL production. *Streptomyces albulus* 11011A was ultimately identified with significantly higher ε‐PL production (2.11 g/L) in the shake flask. By using a two‐stage pH control strategy, this strain accumulated 48.3 g/L of ε‐PL after 192‐hr cultivation in a 5‐L bioreactor, a 10‐fold increase compared to the previous strain (Kahar, Iwata, Hiraki, Park, & Okabe, 2001). Recently, by using an atmospheric and room temperature plasma mutagenesis method, mutant AS3‐14 was obtained with a 66.3% increase in ε‐PL production (Wang, Chen, et al., 2015). In addition, genome shuffling has also proved to be effective, as it was used to combine the positive properties of hyperyield mutants into a fused strain with improved ε‐PL production (Li et al., 2012). Zhou et al. combined genome shuffling and ε‐PL‐resistance mutation to weaken self‐inhibition, and finally enhanced ε‐PL production from 1.80 to 3.11 g/L (Zhou et al., 2015). However, the methods mentioned above were often inefficient or expensive.

Ribosome engineering is a novel and remarkably efficient tool for screening high‐producing strains (Funane et al., 2018; Hu & Ochi, 2001). It can modulate the ribosome by simply inducing mutations that confer resistance to antibiotics (streptomycin, paromomycin, etc.) that attack the ribosome or RNA polymerase, while simultaneously increasing the amounts of target product and protein expression level (Ochi, 2007, 2017). A previous study showed that single drug‐resistance mutation can combine with atmospheric and room temperature plasma mutagenesis or genome shuffling to promote ε‐PL production (Wang et al., 2016). However, these methods were complicated to carry out, and the ε‐PL high‐producing mutants obtained could rarely be further improved. For instance, by utilizing ARTP mutagenesis, we recently induced two antibiotic‐resistance (streptomycin and gentamicin) mutations in strain SG‐31 to promote the ε‐PL synthesis ability. Although a high ε‐PL production of 3.83 g/L was achieved in a shake flask, SG‐31 could barely form spores on plates with other antibiotics (rifamycin, paromomycin, etc.), possibly due to the concentration of drugs used for screening (40–50 MIC of gentamicin). In summary, SG‐31 is not an ideal starting strain for the next phase of breeding. In this study, a simple targeted screening strategy, based on the accumulation of six antibiotic‐resistances in one strain (R6) for the activation of ε‐PL production, was carried out. A high‐throughput screening method was also employed to make the screening procedure more efficient. Finally, a sextuple drug‐resistance mutant R6 was obtained, and a pointed optimization of acidic pH shock strategy was designed for further promotion of ε‐PL production.

## 2 MATERIALS AND METHODS

### 2.1 Microorganism, determination of minimum inhibitory concentration (MIC) and media

The parent strain *Streptomyces albulus* FEEL‐1 was obtained by genome shuffling, as described previously, and preserved in our laboratory (Li et al., 2013). The MICs of streptomycin (Str), gentamicin (Gen), rifampin (Rif), paromomycin (Par), geneticin (Gnt), and lincomycin (Lin) against FEEL‐1 are listed in Table 1.

**Table 1:** Resistance levels of *S. albulus* FEEL‐1 and its derivatives to various antibiotics

	MIC^a^ (μg/ml)						Genotype or description^b^	Mutation introduced at each step	References
	Str	Gen	Rif	Gnt	Par	Lin			
FEEL‐1	3	1	0.2	4	9	20	Original strain	—c	Li et al. (2013)
SG‐31	30	3	0.1	3	12	20	Str^r^ Gen^r^	K108R in ribosomal protein S12	Wang et al., (2017)
R1	9	1	0.2	6	9	20	Str^r^	R99P in ribosomal protein S12	This study
R2	9	3	0.3	6	9	20	Str^r^ Gen^r^	—d	This study
R3	9	3	0.6	4	6	10	Str^r^ Gen^r^ Rif^r^	Q856H in RNA polymerase	This study
R4	9	4	0.8	12	9	10	Str^r^ Gen^r^ Rif^r^ Gnt^r^	—d	This study
R5	9	4	0.8	12	27	10	Str^r^ Gen^r^ Rif^r^ Gnt^r^ Par^r^	—d	This study
R6	9	3	0.5	12	27	60	Str^r^ Gen^r^ Rif^r^ Gnt^r^ Par^r^ Lin^r^	—e	This study

^a^MICs were determined after incubating on BTN agar plate for 72 hr at 30°C. ^b^Strr, Genr, Rifr, Parr, Gntr, and Linr confer resistances to streptomycin, gentamicin, rifampin, paromomycin, geneticin, and lincomycin, respectively. ^c^No mutation: parent strain. ^d^Not determined. ^e^Mutation not detected in ribosomal proteins L14, L15, and S7.

MIC was determined as the lowest concentration of an antibiotic that totally inhibited spore growth after incubation on BTN plates at 30°C for 72 hr. The MIC values of different strains are displayed in Table 1.

The composition of solid medium (BTN) was (g/L) as follows: glucose, 10; yeast extract, 1; and peptone, 2. The composition of the seed and product fermentation medium (YHP/G) was (g/L) as follows: glucose/glycerol, 30/60; (NH_4_)_2_SO_4_, 5; yeast extract, 8; MgSO_4_·7H_2_O, 2; KH_2_PO_4_, 2; FeSO_4_, 0.04; and ZnSO_4_, 0.03. M3G medium was a classical ε‐PL fermentation medium described in the previous literature (Chen et al., 2011).

### 2.2 Culture conditions in microtiter plates, shake flasks, and 5‐L fermenters

Fermentation evaluations were conducted in 24‐deep‐well microtiter plates (5 ml). Spores of mutants were inoculated into the 24‐deep‐well microtiter plates containing 2‐ml distilled YHG medium and then incubated at 30°C at 200 rpm for 96 hr.

Fermentation evaluations in shake flasks were conducted in two steps: For primary screening, three loops of spores from each strain were inoculated in a 250‐ml Erlenmeyer flask containing 40‐ml seed medium for 30 hr. Then, the activated seeds in mid‐exponential phase were inoculated into 250‐ml flasks with an 8% (v/v) inoculum size and incubated at 30°C for 72 hr at 200 rpm. All fermentation tests were performed in three separate shake flasks. During the shake flask fermentation procedure, pH decreased to the lowest value of 3.0 because of (NH_4_)_2_SO_4_ consumption and some uncertain organic acid production. When pH was below the optimal pH of 4.0, cell growth and ε‐PL synthesis were suppressed, which contributed to the low ε‐PL production (only a few grams) in the shake flask. For further evaluation of the potential advantages of mutants, batch and fed‐batch fermentations were performed in 5‐L stirred tank bioreactors (Baoxing, Shanghai, China).

In batch and fed‐batch fermentations, four loops of spores from each strain were inoculated into 500‐mL shake flasks and incubated. After 30 hr of cultivation, 240‐mL precultured seed was cultivated at 30°C in a 5‐L fermenter containing 3.2‐L aseptic fermentation medium (initial pH 6.8), and NH_3_·H_2_O solution was added automatically to maintain pH 4.2 for ε‐PL synthesis before the glucose was depleted. In this study, two types of fed‐batch fermentation strategies, the constant pH control strategy and acid pH shock strategy, were applied to evaluate the fermentation performances of the strains used. The constant pH control strategy is quite similar to batch fermentation. Glucose concentration was maintained below 10 g/L by automatic pulsing using a peristaltic pump connected to a sterile 85% (w/v) glucose solution. NH_3_‐N concentration was maintained below 1 g/L by periodically adding 600 g/L aseptic (NH_4_)_2_SO_4_ solution. The acid pH shock strategy was conducted in three steps, as described by Ren, Chen, et al. (2015), with some modifications: (a) Pre‐acid‐shock adaption phase: pH was set to 5.0 to lighten the damage caused by the followed pH shock; (b) pH shock phase: when pH dropped to the lowest value of 3.3, it was maintained at that value for 12 hr; and (c) recovery phase: pH was control at 4.2 to provide the optimal environment for the strain to produce ε‐PL.

### 2.3 Strain selection by cumulative drug‐resistance mutations and high‐throughput screening

**Figure 1:**
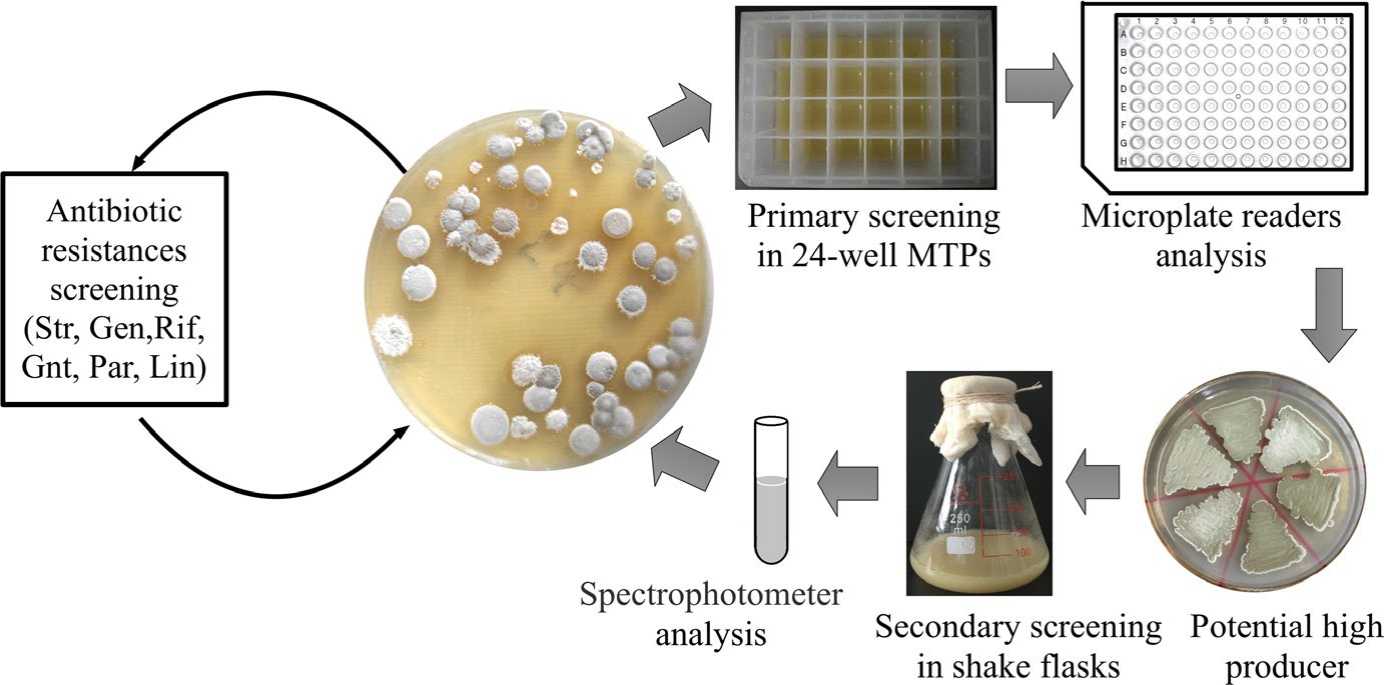
Illustration of the procedure used for the sequential introduction of drug‐resistance mutations to generate the multiple‐drug‐resistance mutants

*S. albulus* FEEL‐1 was the initial strain used to select for multiple‐drug‐resistance mutants. The spores of FEEL‐1 were incubated on BTN agar plates at 30°C for 7 days. After the spore maturation, the fresh spore suspension (10^8^ CFU/ml) of FEEL‐1 was spread on BTN agar plate with 3‐fold Str concentration compared with the parental strain's MIC, and cultivated for approximately 7–10 days for sporulation. The single drug‐resistance strains obtained were randomly selected and sequentially evaluated in 24‐deep‐well microtiter plates and shake flasks (Figure 1). The mutant with the highest ε‐PL production was named R1 and was utilized as the starting strain for the next round of screening. In the second round of breeding, a spore suspension of R1 was spread on BTN agar plates containing 3 MIC of Gen and the double‐resistance mutant R2 with the highest ε‐PL production was also selected using microtiter plates. All of the mutations arose spontaneously, and no induced mutagenesis was required. All mutants with the highest ε‐PL production were kept for the next round of screening. Illustration of the sequential screening procedure is shown in Figure 1. Finally, following the screening steps for resistance to Rif, Par, Gnt, and Lin, the triple‐, quadruple‐, quintuple‐, and sextuple‐resistance strains (R3, R4, R5, R6) were finally obtained and used in the experiments described in Sections 3.2 and 3.3.

### 2.4 Evaluation of fermentation performance in 5‐L bioreactors by average ε‐PL productivity and average ε‐PL formation rate (*q*_*p*_)

ε‐PL productivity (g/L/day) and *q*_*p*_ (day^−1^) were calculated by the following formulas:

$$\varepsilon -\text{PL}\;\text{Productivity}\;=\;\frac{\Delta P}{\Delta t}\;=\;\frac{P_{t2}-P_{t1}}{t2-t1}$$

$$\overset{¯}{q_{p}}\;=\;\frac{1}{\overset{¯}{x_{t2}}}\;=\;\frac{\Delta P}{\Delta t}\;=\;\frac{1}{\frac{1}{2}\left(x_{t1}\;+\;x_{t2}\right)}\;\times \;\frac{P_{t2}-P_{t1}}{t2-t1}$$

where *x* is dry cell weight (DCW), *p* is ε‐PL concentration, and *t* is fermentation time.

### 2.5 Difference in colony morphology

Following plate cultivation, the colony morphologies of FEEL‐1 and its derivatives were visually observed and photographed. Strain mycelia were then prepared for high‐resolution scanning electron microscopy (SEM) analysis as described (Cho et al., 2017).

### 2.6 Intracellular metabolite analysis, enzyme analysis, and transcription analysis

For the intracellular ATP assay, mycelia pellets were obtained by centrifugation at 3,600 × g for 10 min and washed twice with distilled water. Then, mycelia pellets were suspended in 0.5 M HClO_4_ at 0°C and ultrasonicated for 10 min with 3 s of running and 1‐s intervals. The debris was removed by centrifugation at 12,000 × g for 20 min. The supernatant was filtered with a 0.22‐μm membrane, and ATP concentration was determined by HPLC (Agilent 1200, USA) using a LAChrom C18‐AQ column (250 × 4.6 mm) and a spectrophotometer at 254 nm at 25°C. The mobile phase contained the following: 5% acetonitrile, 95% 0.2 M Na_2_HPO_4_‐NaH_2_PO_4_ buffer (pH 7.0), and 10 mM tetrabutylammonium bromide, with the flow rate set to 1.0 ml/min. Assays were performed in triplicate at 4°C.

For the intracellular Lys assay, 1‐mL broth culture was centrifuged at 7000 ×  g for 1 min, washed twice with distilled water, and resuspended in 1 mL of 10% trichloroacetic acid at 37°C for 10 min. Subsequently, the sample was boiled for 30 min and centrifuged at 12,000 × g for 20 min to discard the cell debris. The resultant supernatant was analyzed with HPLC as described (Fountoulakis & Lahm, 1998). Assays were performed in triplicate.

To measure the metabolic enzymes, mycelia pellets were obtained by centrifugation at 3,600  ×  g for 10 min, washed twice with 0.2% KCl, and suspended in 100 mM Tris–HCl buffer (pH 7.5) containing 20% glycerol and 1 mM dithiothreitol (DTT). Samples were immediately processed by ultrasonication in an ice bath for 30 min of 2‐s running and 2‐s intervals with a 650‐W sonicator (SM‐650D; Shunma Tech., Nanjing, China). Unbroken cells and debris were then removed by centrifugation at 12,000 × g for 20 min. The supernatant was used as the cell extract for the immediate determination of enzyme activities. The assays of glucose‐6‐phosphate dehydrogenase, pyruvate kinase, aspartokinase, and ε‐poly‐lysine synthetase were examined, as described previously (Zeng et al., 2014). All enzymes assays were carried out at 30°C. Protein concentration of cell extraction was measured using the Super‐Bradford Protein Assay Kit. All the enzyme assays were conducted in triplicate.

RNAs from different strains were extracted from the mycelia utilizing a MiniBEST Universal RNA Extraction Kit (Thermo Fisher, Waltham, MA, USA) according to the provided instructions. A spectrophotometer ND‐1000 (Thermo Fisher, Waltham, MA, USA) was used to determine the quality and quantity of RNAs. Transcription reactions were performed with a Prime Script RT Reagent Kit, and transcription profiles of ε‐PL synthetase gene *pls* (Gene ID: 343488678) were determined using qRT‐PCR with a Dongsheng qRT‐PCR kit and mixing 2 × SYBR Green Mix (8.5 μL), forward primer (5 μM), reverse primer (5 μM), RNase‐free H_2_O (6.4 μL), and cDNA (1.25 μL). A final volume of 17 μL was prepared by adding RNase‐free H_2_O. Amplification was performed in a Cfx96 Real‐Time PCR system. The conditions of qRT‐PCR were as follows: 50°C for 2 min, 95°C for 10 min, followed by 40 two‐temperature cycles (95°C for 15 s and 60°C for 1 min). All of these experiments were performed in triplicate by using RNA samples extracted from three independent cultures. The qRT‐PCR result analyses were subjected to the $2^{-(\Delta \Delta C_{t})}$ method for relative quantification with *16S rRNA* as the endogenous control gene (Livak & Schmittgen, 2001). The design of the *pls* forward (5′‐TGCCGAGCGTGAAATTGTGA‐3′) and reverse (5′‐CGTGAATGCCAGCAGCCTCCA‐3′) primers was based on the genome sequence of *S. albulus* PD‐1 (GenBank: AXDB02000025.1). All procedures were repeated three times and conducted at 0–4°C.

### 2.7 Analytical methods

The broth cultures obtained from 24‐deep‐well microtiter plates were centrifuged (1,3000 × g,10 min) and the precipitates washed twice and vacuum‐dried at 65°C to a constant weight in order to evaluate DCW. The broth cultures obtained from the shake flasks or 5‐L fermenters were sampled and centrifuged (4,500 × g, 10 min). Then, DCW was measured gravimetrically by filtering the sediment, washing twice with distilled water, and drying at 100°C until constant weight was achieved.

The resulting supernatant was used to determine the concentrations of glucose, nitrogen, and ε‐PL. Glucose concentration was detected with a biosensor analyzer SBA‐40D (Shandong Academy of Sciences). Nitrogen concentration was detected with a method described by Lu et al. (2011). ε‐PL concentration was detected according to a method described by Itzhaki (Itzhaki, 1972) with some modifications. For example, with use of an eight‐channel pipette (Eppendorf Xplorer plus, Hamburg, Germany), 50 μL of the supernatant in each well of the 24‐well microtiter plates was transferred to a 96‐deep‐well microtiter plates and mixed with 950 μL of phosphoric acid buffer. Then, 500 μL of diluted supernatants was transferred to another 48‐deep‐well microtiter plates containing 500 μL of methyl orange (MeO). The mixtures were vortexed and incubated at 30°C for 30 min at 200 rpm. The plate was then centrifuged (1600 × g, 25 min), and 10 μL of the resulting supernatant was transferred into a 96‐deep‐well microtiter plates containing 190 μL phosphoric acid buffer. The absorbance of the diluted supernatants was evaluated at 465 nm using a Multiskan FC microplate reader (Thermo Fisher, Waltham, MA, USA).

## 3 RESULTS

### 3.1 Adequate screening dosage of Str, Gen, Rif, Gnt, Par, and Lin on *S. albulus* FEEL‐1

**Figure 2:**
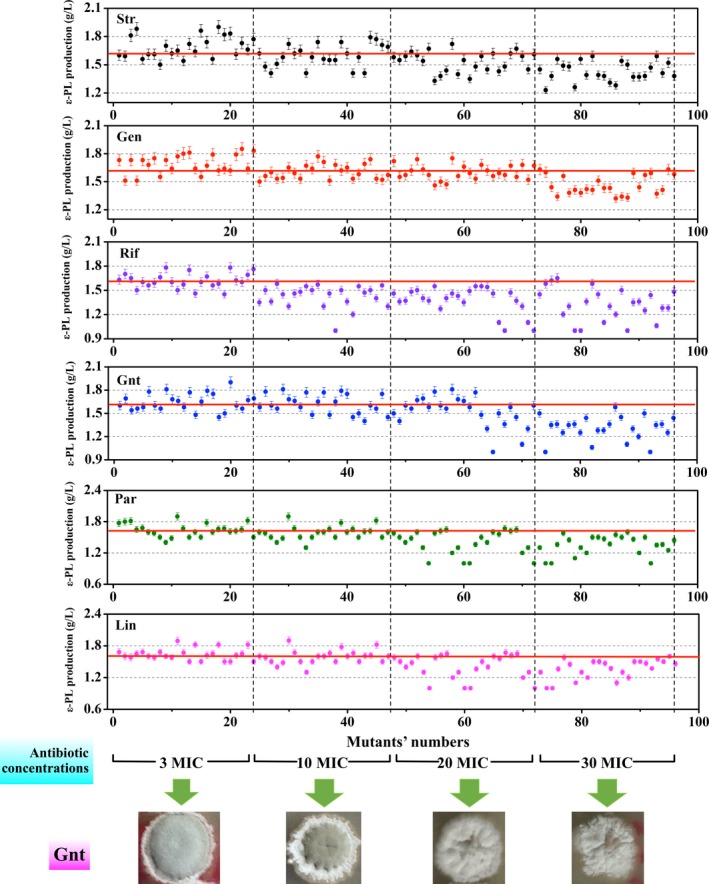
ε‐PL production of mutants on various concentrations of Str, Gen, Rif, Gnt, Par, and Lin. Fermentation assays to determine adequate screening concentrations of Str, Gen, Rif, Gnt, Par, and Lin were conducted by incubating FEEL‐1's spores on BTN agar plate with 3 MIC, 10 MIC, 20 MIC, and 30 MIC of Str, Gen, Rif, Gnt, Par, and Lin, respectively. The ε‐PL production rates of the obtained mutants were evaluated in 24‐well microtiter plates

In a recent study, gradient plates (antibiotic concentrations: 0–50 MIC) were utilized to achieve double‐resistance mutants (Wang et al., 2017). However, after preservation for 2 months, we found that the genetic stability of the mutants selected from areas of higher drug concentration was much lower than those from areas of lower drug concentration, as manifested by the fall in ε‐PL production and antibiotic resistance. Some reports have also indicated that antibiotic dosage significantly influences mutation efficiency and positive mutation rate in the ribosome engineering procedure (Ochi et al., 2004). Thus, we designed an experiment to use moderate concentrations of six antibiotics (Str, Gen, Rif, Gnt, Par, and Lin) for ε‐PL‐producing *Streptomyces albulus*. In this assay, a spore suspension of original strain FEEL‐1 was separately spread on to plates containing varied concentrations (3‐, 10‐, 20‐, 30‐fold of the parental strain's MIC) of the six antibiotics to evaluate their effect on ε‐PL production. According to Figure 2, mutants tended to exhibit higher ε‐PL production when selected on plates of lower drug concentration (3 or 10 MIC). As drug concentrations reached 30 MIC, the ε‐PL produced by some mutants was found to be only around 1.0–1.2 g/L, even lower than the FEEL‐1 (1.6 g/L). Consequently, the highest ε‐PL produced by the colonies (separately containing Str^r^, Gen^r^, Rif^r^, Gnt^r^, Par^r^, and Lin^r^) was 1.91, 1.82, 1.85, 1.78, 1.90, and 1.89 g/L, respectively, which were 19.4%, 13.8%, 15.6%, 11.2%, 18.7%, and 18.1% higher than FEEL‐1. More importantly, five out of these six high‐producing colonies were screened from plates with 3 MIC of each antibiotic. Furthermore, as antibiotic concentrations increased, positive mutation rate and mutation efficiency gradually declined (data not shown). In addition, mutants on plates grew more slowly and produced less aerial mycelia and spores, and transferring these mycelia to a drug‐free plate does not solve the problem. This result can directly contribute to the decline of propagating capacity and genetic stability. Finally, we chose 3 MIC of each antibiotic as the ideal concentration for strain evolution.

### 3.2 Construction of multiple antibiotic‐resistance mutants

Starting with FEEL‐1, we screened for the Str^r^ mutant by directly spreading a spore suspension on BTN plates with 3 MIC of Str. After culturing for 8 days, 100 colonies that showed more rapid growth rates were picked out, and approximately 19% of them displayed enhanced ε‐PL production compared with FEEL‐1. The highest production mutant was named R1, and it produced 1.93 g/L ε‐PL in a shake flask, 20.6% higher than FEEL‐1. Subsequently, successive screenings were conducted with R1 to construct double, triple, quadruple, quintuple, and sextuple mutants (R2, R3, R4, R5, R6). The period required for all screening procedures was approximately 7 months. Eventually, the sextuple‐resistance mutant R6, with a significantly improved ε‐PL production (4.41 g/L), 2.75‐fold compared to FEEL‐1 in the shake flask, was considered to be the preferable ε‐PL producer for subsequent assays. During the screening process, 5%–25% of mutants produced more ε‐PL than their parent strains, proving the high efficiency of acquiring high‐producing colonies by cumulative low‐concentration resistance of six drugs.

**Figure 3:**
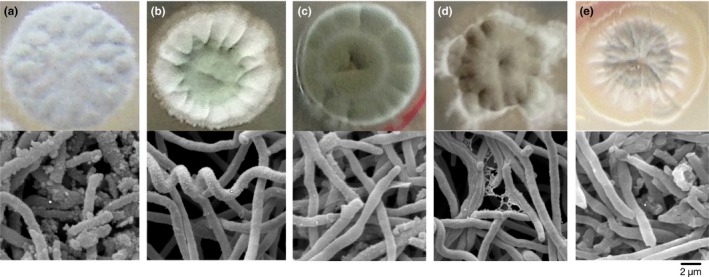
Culture dish photographs and SEM micrographs of the original strain FEEL‐1 and drug‐resistance mutants. Strains were incubated on BTN medium at 30°C for 8 days. (a) FEEL‐1 (b) R2 (c) R4 (d) R6 (e) SG‐31

Mutant morphologies changed during the screening procedure (Figure 3). The colors of the spores were gradually changed from light green to dark green (from FEEL‐1 to R4), then transformed to dark brown (R6). When making the spore suspension, we found that the spore shapes of FEEL‐1 and R2 were stuck together in a lamellar shape, while those of R4, R6, and SG‐31 were nonsticky and granular. On the other hand, mycelia morphologies between FEEL‐1 and its derivatives also showed some differences when observed by scanning electron microscopy. One prominent feature is the visible small, pointy projections on the surface of the FEEL‐1 hypha. Along with the elevated ε‐PL production, these pointy projections gradually disappeared, particularly in R6 and SG‐31.

### 3.3 ε‐PL overproduction and mutation analysis

**Figure 4:**
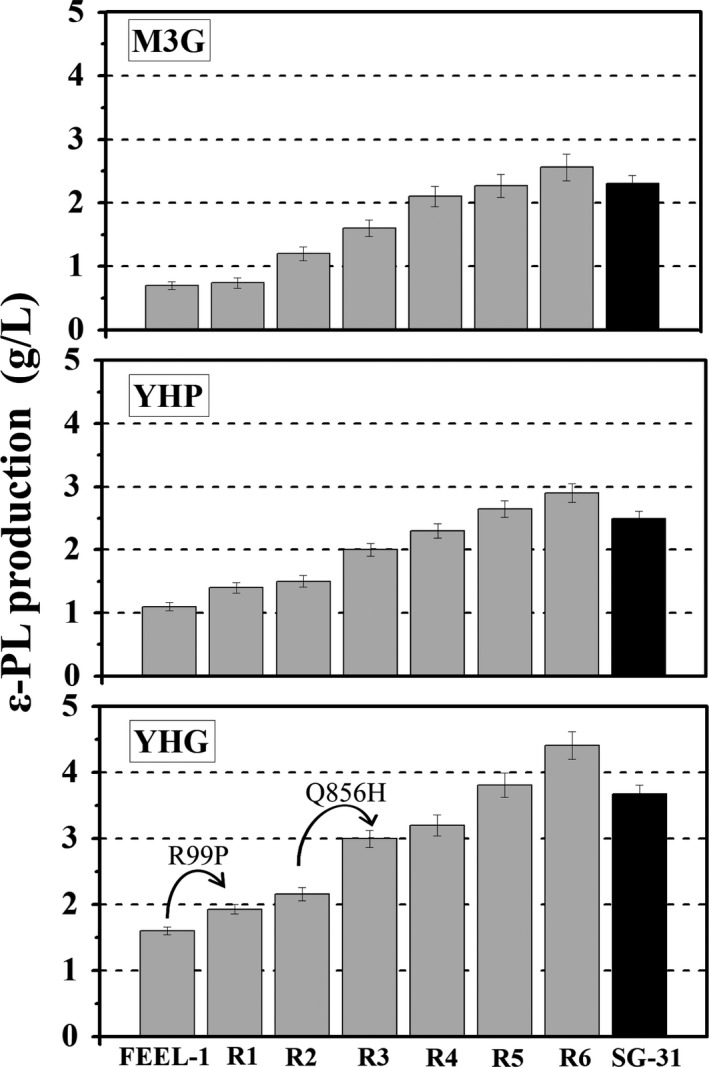
Evaluation of ε‐PL production in liquid culture from the strains. Spores of strains were separately inoculated into M3G, YHP, and YHG medium and incubated at 30°C for 4 days

For further evaluation of fermentation performance, the original strain (FEEL‐1) and the mutants (R1‐R6 and SG‐31) were cultivated in three different ε‐PL fermentation media (M3G, YHP, and YHG). The relationship between ε‐PL production and the number of drug‐resistance mutations in each strain is shown in Figure 4. As expected, R6 had the highest ε‐PL production in all three‐fermentation media, proving that the ability of R6 to synthesize ε‐PL is actually improved. M3G medium is a common medium reported in many studies (Chen et al., 2011; Hu & Ochi, 2001), and cultivation in this medium helped us to compare mutant fermentation performances with other reported strains. In M3G medium, R5 and R6 produced 2.27 g/L and 2.56 g/L ε‐PL, 3.24‐ and 3.65‐fold, respectively, compared to that of FEEL‐1 (0.7 g/L). YHP medium was an optimized medium based on the M3G medium for FEEL‐1. In YHP medium, ε‐PL production of R6 was 2.9 g/L, 163% higher than FEEL‐1 (1.1 g/L). Enhanced ε‐PL production was more pronounced when FEEL‐1 and its derivatives were cultivated in YHG medium (glycerin is substituted for glucose as the carbon resource in YHG medium). In this medium, R5 and R6 produced 3.81 g/L and 4.41 g/L ε‐PL, respectively, approximately 2.38‐ and 2.75‐fold compared to the 1.6 g/L produced by FEEL‐1. More significantly, R6 was genetically stable, and no apparent change was found in morphology, ε‐PL production, or DCW, even after sequentially subculturing on BTN agar plates for eight generations (Supporting information Table S1).

Previously, we found that a Str^r^ mutation (K108R) in ribosomal protein S12 effectively led to ε‐PL overproduction. Hence, we sequenced ribosomal protein S12 encoding gene *rpsL* (Gene ID: 1100100) in Str^r^ mutants. To our surprise, R1 with an improved ε‐PL production possessed a novel R99P amino acid substitution in S12 (Table 1), besides K108R. Furthermore, we also sequenced RNA polymerase encoding gene *rpoB* (Gene ID: 1100095) and found a Q856H mutation site in a RNA polymerase subunit. Interestingly, the new mutation in S12, plus the mutation in the RNA polymerase subunit, enabled cells to produce more ε‐PL, proving the feasibility of accumulating multiple antibiotic‐resistance mutations for strain evolution. In addition, the introduction of a mutation in the RNA polymerase subunit (Rif^r^ mutation) increased R3 sensitivity to Gnt and Lin, while the unknown Lin^r^ mutation increased R6 sensitivity to Gen and Rif (Table 1).

### 3.4 Optimization of pH shock parameters and evaluation of fermentation performance of R6

**Figure 5:**
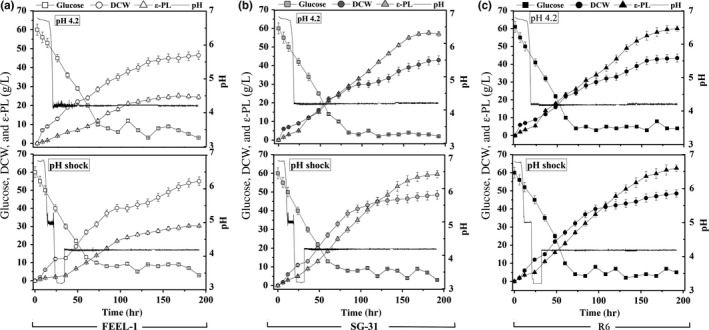
Fed‐batch fermentation performance of strains utilizing two different strategies. Strains (a, FEEL‐1; b, SG‐31; c, R6) were separately cultured in 5‐L bioreactors using two strategies (constant pH control strategy or original pH shock strategy, as described in Section 3) at 30°C for 192 hr

Two effective strategies, “constant pH control” and “acidic pH shock” (Ren, Chen, et al., 2015) were applied to evaluate the fermentation performance of R6 (Figure 5). By using a constant pH control strategy, R6 achieved 59.7 g/L of ε‐PL, 2.37‐fold greater than that of (25.2 g/L) FEEL‐1 (Table 2). The ε‐PL productivity and *q*_*p*_ of R6 were also higher than those of FEEL‐1. Results of the acidic pH shock strategy were analogous to the constant pH control strategy, and ε‐PL production of R6 improved to 62.5 g/L, about twice as much as FEEL‐1 (30.3 g/L). Surprisingly, although R6 produced more ε‐PL in the shake flask than SG‐31, the difference in ε‐PL production between the strains was not as high as we expected. The ε‐PL productivity and the *q*_*p*_ of R6 were even lower than that of SG‐31 using the constant pH strategy.

**Table 2:** Fermentation performances of FEEL‐1, SG‐31, and R6 with two pH control strategies

Strategy	Strain	*T* a (hr)	ε‐PL production^b^ (g/L)	DCW (g/L)	*q*_*p*_ c (1/day)	ε‐PL productivity (g/L/day)
Constant pH control	FEEL‐1	168	25.2 ± 1.2	45.1 ± 1.8	0.16	3.60 ± 0.16
	SG‐31	174	57.6 ± 1.3	41.6 ± 2.2	0.38	7.94 ± 0.17
	R6	192	59.7 ± 1.5	43.5 ± 1.7	0.34	7.46 ± 0.18
Acidic pH shock	FEEL‐1	180	30.3 ± 0.4	54.1 ± 2.4	0.15	4.04 ± 0.05
	SG‐31	192	59.5 ± 1.2	48.5 ± 1.8	0.31	7.44 ± 0.15
	R6	192	62.5 ± 1.8	47.8 ± 1.4	0.33	7.81 ± 0.22

^a^Time for strains to reach their maximum ε‐PL production. ^b^Maximum ε‐PL production of each strain. ^c^ *q*_*p*_ is the ε‐PL formation rate of each fed‐batch fermentation.

The adaption pH value and time of the R6 mutant may be altered in the process of acquiring multiple antibiotic‐resistance mutations, suggesting that the pre‐acid‐shock adaption conditions should be redesigned. Firstly, the adaption pH values were separately controlled at 6.5, 5.5, and 4.5, and the time at which DCW doubled was recorded (Supporting information Figure S1a,b). A 12‐hr pH shock was then conducted after each pre‐acid‐shock adaption mode (Supporting information Figure S1c). After 54 hr of culture, ε‐PL production in cultures subjected to 6.5 and 5.5 preadaption pH modes was 7.3 and 7.1 g/L (Supporting information Figure S1d), much higher than preadaption at pH 4.5 (5.9 g/L). However, *q*_*p*_ obtained at a 6.5 preadaption pH was lower than that at 5.5 preadaption pH (data not shown). Finally, we considered 5.5 to be the best pH for preadaption. Subsequently, three different time periods (5, 10, and 15 hr) were maintained in a sequence of batch fermentation assays to determine the optimal pH shock interval (Supporting information Figure S2). The results of cell growth, *q*_*p*_, ε‐PL production, and productivity are displayed in Table 3. Analysis of the results showed that after 5 hr of pH shock, the cells continued to grow and produce ε‐PL. Conversely, after suffering from 10 or 15 hr of pH shock, there was a long lag phase before the cells regained the ability to grow and synthesize ε‐PL. Moreover, after pH shock, and in spite of the lower ε‐PL production, the ε‐PL productivity and *q*_*p*_ of cells exposed to 5‐hr pH shock were much higher than those exposed to 10‐ and 15‐hr pH shock (Table 3). In conclusion, 5 hr was the ideal pH shock interval for improving R6′s ε‐PL synthesis ability. Considering the above results, the optimized acidic pH shock strategy was as follows: (a) Pre‐acid‐shock adaption phase: pH was controlled at 5.5 for 10 hr to introduce an acid tolerance response. (b) Acidic pH shock phase: pH naturally declined from 4.0 to 3.3 and was maintained for 5 hr for activation of ε‐PL synthesis. (c) Recovery phase: pH was set at 4.2 until the end of fermentation.

**Table 3:** Fermentation performance at different pH shock intervals of mutant R6

pH shock interval (hr)	*T* (hr)	*T* a (hr)	ε‐PL production (g/L)	DCW (g/L)	*q*_*p*_ b (1/day)	ε‐PL productivity^c^ (g/L/day)
5	48	14.5	4.0 ± 0.2	21.1 ± 1.1	0.31	4.96 ± 0.23
10	65	26.5	4.9 ± 0.1	19.5 ± 1.3	0.26	3.80 ± 0.18
15	74	30.5	4.6 ± 0.2	18.8 ± 0.8	0.22	3.07 ± 0.15

^a^Time since ε‐PL production began to increase after pH shock. ^b^ε‐PL formation rate after pH shock. ^c^Average ε‐PL productivity after pH shock.

FEEL‐1, SG‐31, and R6 were compared again using the optimal acidic pH shock strategy (Supporting information Figure S3), and the results are shown in Table 4. After utilizing the optimized pH shock strategy, R6 accumulated 70.3 g/L of ε‐PL, 10.8 g/L higher than the previously reported ε‐PL production of SG‐31. A variety of strains for which ε‐PL production has been measured are compared in Table 5. To the best of our knowledge, R6 has achieved the highest reported ε‐PL production. In summary, after ribosome engineering and acidic pH shock optimization, R6 produced 2.79‐fold more ε‐PL than the original strain FEEL‐1.

**Table 4:** Fermentation performance of FEEL‐1, SG‐31, and R6 using the new pH shock strategy

Strategy	Strain	*T* (hr)	ε‐PL production (g/L)	DCW (g/L)	*q*_*p*_ (1/day)	ε‐PL productivity (g/L/day)
New acidic pH shock	FEEL‐1	180	39.2 ± 1.8	64.1 ± 2.2	0.16	5.21 ± 0.24
	SG‐31	192	63.1 ± 2.1	54.2 ± 2.5	0.29	7.90 ± 0.26
	R6	204	70.3 ± 2.4	48.5 ± 2.3	0.33	8.01 ± 0.28

**Table 5:** Summary of fed‐batch fermentation performance of reported strains using different strategies

ε‐PL‐producing strain	Fermentation time (day)	Strategy	ε‐PL production (g/L)	DCW (g/L)	ε‐PL productivity (g/L)	References
*S. albulus* S410	8	Two‐stage pH control^a^	48.3	27	6.04	Kahar et al. (2001)
*S. albulus* IFO 14147	10	pH 4.0^b^	5.16	34.21	0.52	Shih et al. (2006)
*Kitasatospora* sp. MY 5–36	3.7	Cell immobilization	34.11	22.98	9.22	Zhang, Feng, Xu, Yao, and Ouyang (2010)
*S. griseofuscus*	5	pH 3.5	7.5	17	1.5	Li et al. (2011)
*S. albulus* FEEL‐1	7	pH 3.8	24.5	25.88	3.50	Li et al. (2013)
*S. albulus* NK660	9.1	pH 4.0	4.2	17	0.46	Geng et al. (2014)
*S. albulus* PD‐1	7	pH 4.0 + Citric acid^c^	29.7	33	4.24	Xia et al. (2014)
*S. albulus* M‐Z18	8	acidic pH shock^d^	54.7	76.35	6.84	Ren, Chen, et al. (2015)
	8	Acidic pH shock+ Talc^e^	62.36	78.13	7.80	Ren, Xu, et al. (2015)
*S. albulus* SG‐31	7.25	pH 4.2	59.5	46.1	8.21	Wang et al. (2017)
*S. albulus* R6	8.5	New acidic pH shock^f^	70.3	48.5	8.27	This study

^a^In this strategy, pH was set in two phase: (a) pH 5.0 for cell growth and (b) pH 4.0 for ε‐PL production. ^b^pH was maintained at 4.0 during the whole fermentation process. ^c^In the fermentation process, pH was maintained at 4.0 and citric acid was added to increase ε‐PL production. ^d^Representative for the acid pH shock strategy. ^e^Microparticles were added to enhance ε‐PL production while using acid pH shock strategy. ^f^The new pH shock strategy obtained in this study for further activation of ε‐PL synthesis.

### 3.5 Transcription analysis, enzyme analysis, and intracellular metabolite analysis

**Figure 6:**
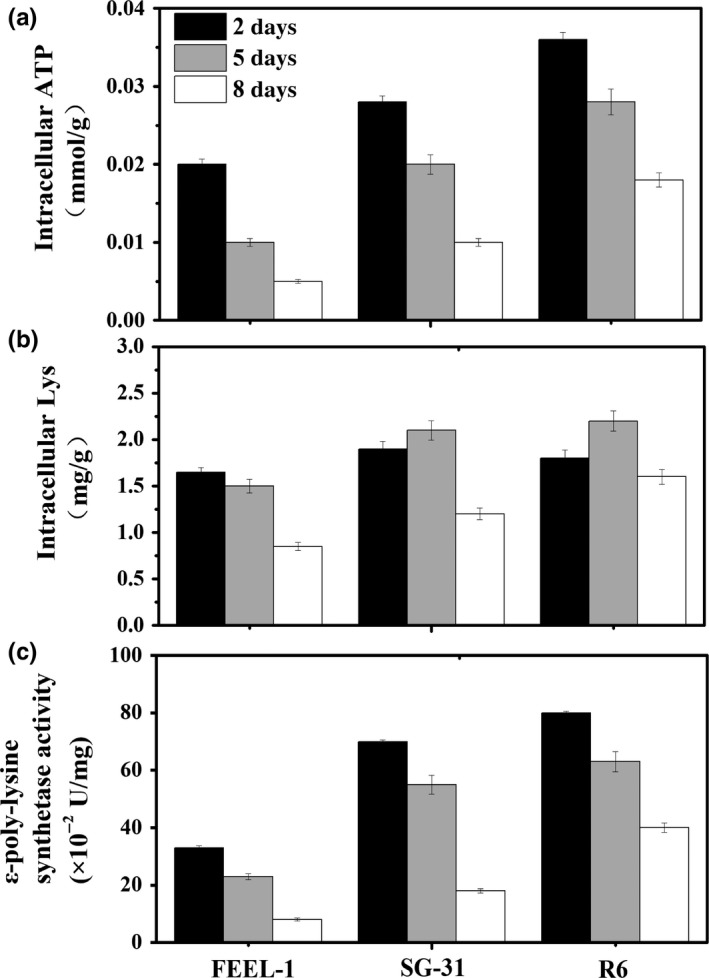
Comparison of ε‐PL synthetase activity, intracellular ATP, and Lys concentrations of strains (FEEL‐1, SG‐31, and R6) in early (2 days), middle (5 days), and late phases (8 days) of the fed‐batch fermentations. All assays were repeated three times, and error bars represent the standard deviation

**Figure 7:**
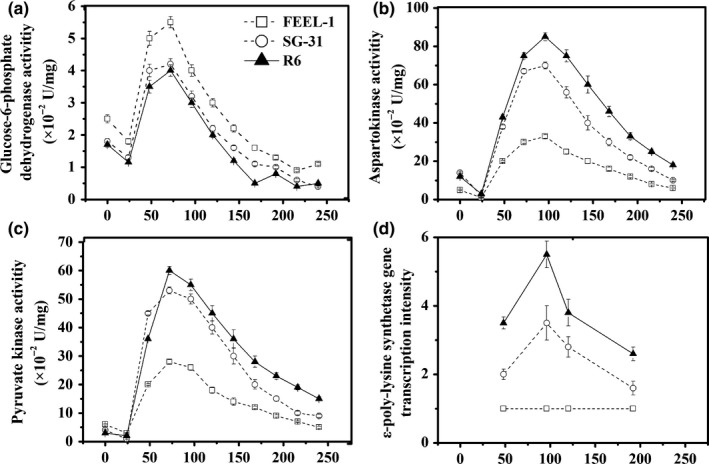
Comparison of key enzyme activities and the *pls* transcription levels of different strains during the fed‐batch fermentations. All assays were repeated three times, and the error bars represent the standard deviation

In comparison with FEEL‐1, the ε‐PL synthetase activity, intracellular ATP, and Lys concentrations of R6 were enhanced in the middle and late phases (5 and 8 days) of fermentation (Figure 6), which is the direct reason for the prolonged time (204 hr) of ε‐PL efficient synthesis and ε‐PL overproduction. Since pyruvate kinase and aspartokinase are the key enzymes of the glycolytic pathway (EMP) and L‐lysine synthetic pathway (DAP), the elevated precursor (Lys) concentrations can be expounded by the improved activities of pyruvate kinase and aspartokinase during the whole fed‐batch fermentation process (Figure 7b,c). On the other hand, the ε‐poly‐lysine synthetase gene (*pls*) transcription level of R6 was much higher than that of FEEL‐1 (Figure 7d), which can be responsible for the improved ε‐PL synthetase activity (Figure 6c). As shown in Figure 7a, glucose‐6‐phosphate dehydrogenase activities of the three strains increased immediately following pH shock and then decreased after 72‐hr culture, which was consistent with their slow cell growth rates in the late fermentation phase. Moreover, glucose‐6‐phosphate dehydrogenase activity of R6 was a little lower than that of SG‐31, which may be one reason for the R6's lower DCW when compared to SG‐31.

## 4 DISCUSSION

In previous studies, screening with low antibiotic concentrations was seldom used in ribosome engineering. Researchers tended to choose higher antibiotic concentrations (20–50 MIC) to select the strains with mutations in ribosome gene *rpsL* or *rplF*, which further contributed to the aberrant protein synthesis activity and high production (Wang, Hosaka, & Ochi, 2008). Some studies also showed a positive correlation between antibiotic concentration and target product production (Zhu et al., 2018). However, we concluded from our previous study that colonies cultured on plates containing lower antibiotics exhibited better growth states and higher ε‐PL production. Therefore, in this study, we considered that low drug concentrations provide the best conditions for breeding. This result is similar to a previous report on the mutation caused by low‐level streptomycin resistance which can result in an antibiotic overproduction in *Streptomyces coelicolor* A3(2) (Nishimura, Hosaka, Tokuyama, Okamoto & Ochi, 2007). Although causal relationship between the increased ε‐poly‐lysine production and each drug‐resistance mutation was not demonstrated in the present work due to the lack of feasible genetic system in this organism, high‐frequency appearance of high‐producing strains among the drug‐resistance mutants (Figure 2) shows a likely causality of these events.

Studies on antibiotic action have demonstrated that many antibiotics target ribosomal components (Wirmer & Westhof, 2006). For instance, most Str^r^ and Par^r^ mutations are clustered in the *rpsL* gene, but it has also been reported that low‐level Str^r^ may result in some mutations in the *rsmG* gene (Nishimura, Hosaka, Tokuyama, Okamoto, & Ochi, 2007; Shima, Hesketh, Okamoto, Kawamoto, & Ochi, 1996). Gen^r^ frequently occurs due to mutations in ribosomal protein L6, and Lin^r^ is often due to mutations in ribosomal proteins S7, L14, and L15 (Hummel, Piepersberg, & Bock, 1979). Sextuple‐resistance mutant R6, however, did not exhibit mutations in the L6‐encoding gene *rplF*, or the genes encoding S7, L14, and L15. These results indicate that some unknown novel types of mutations (possibly in ribosomes) account for multiple‐drug resistance and ε‐PL overproduction.

In general, the level of production achieved in the shake flask positively correlated with that of the bioreactor. However, compared to SG‐31, R6 had few advantages in the 5‐L bioreactor fermentation process in spite of higher ε‐PL production in the shake flask. Acidic pH shock was then optimized to stimulate strains (FEEL‐1, SG‐31, and R6) to overproduce ε‐PL. According to the result, we concluded that the pH shock strategy had a more profound influence on the parent strain (FEEL‐1) than the mutants (R6) because ε‐PL production of FEEL‐1 rose the most (a 55.6% of enhancement). This result was similar to wild‐type *Streptomyces albulus* M‐Z18, which had a 52.5% promotion in ε‐PL production by utilization of the original pH shock strategy (Ren, Chen, et al., 2015). Despite this, R6's production advantage was fully developed under the effect of the new pH shock strategy. Finally, R6 achieved an enhanced ε‐PL production of 70.3 g/L, significantly higher than those of FEEL‐1 and SG‐31, proving the effectiveness and feasibility of the optimization. In our previous study, when designing the pH shock strategy, more attention was given to ε‐PL production and productivity than *q*_*p*_ and the amount of DCW. This may be one reason for the undue high DCW (almost 80 g/L) of *S. albulus* M‐Z18. Excessive level of DCW brings about high viscosity of the liquid culture, which may affect the dissolved oxygen rate. Hence, we mainly focused on ε‐PL formation rate (*q*_*p*_) when optimizing the acidic pH shock strategy to avoid the result of high ε‐PL production with undue high DCW.

Our ultimate target is to acquire a mutant with relatively high ε‐PL and low DCW for industry needs. R6's DCW is much lower than FEEL‐1, which means that every R6 cell can synthesize more ε‐PL. Moreover, the relative decrease in biomass can reduce the workload of the purification of ε‐PL to some extent. This result resembles that of our previous report that DCW of double‐resistance strain SG‐31 was lower than FEEL‐1, which means the multiple drug‐resistance mutants share the same characteristic of relatively low DCW and high ε‐PL production (Wang et al., 2017). Conversely, as the number of mutations increased, the spore growth rates of the mutants (from R1 to R6) on plates decreased (data not shown). These findings can be explained by the fact that drug resistance was often acquired at the cost of growth fitness (Andersson & Levin, 1999).

Some researchers found that the mutant ribosomes (caused by accumulated drug resistance) can contribute to aberrant protein synthesis activity and antibiotic overproduction (Hu & Ochi, 2001). Based on this theory, we concluded that the mutations in ribosome and RNA polymerase genes of sextuple mutant R6 may cause the enhancement of pyruvate kinase, aspartokinase, and ε‐PL synthetase activities. Moreover, ε‐PL synthetase is one of the most important enzymes in the ε‐PL synthesis pathway, as it can directly synthesize ε‐PL from monomer Lys by consumption of ATPs. Perhaps some unknown regulation of protein synthesis is also activated by ribosome engineering, which further results in an increase in *pls* transcription (Figure 7d), ε‐PL synthetase activity, and ε‐PL production.

Overall, we have succeeded in isolating a ε‐PL high‐producing strain R6 that accumulated six antibiotic‐resistance mutations. This method can also be utilized for improving production of target products in other microorganisms. After optimization of the fermentation process, a new and optimal acidic pH shock method was especially established, indicating that R6 could be a promising producer for large‐scale ε‐PL production.

## CONFLICT OF INTEREST

The authors declare no conflict of interest.

## AUTHORS’ CONTRIBUTIONS

L.W designed experiments and wrote the manuscript. L.W, L.S, J.Z, and Y.L carried out experiments. L.W analyzed experimental results. X.C, T.L, and Z.M assisted with intracellular metabolite analysis and transcription analysis.

## ETHICS STATEMENT

This article does not contain any studies with human or animals performed by any of the authors.

## Data Availability

All data generated or analyzed during this study are included in this published article.
