# Supplementary figures and images for: Efficiently activated ε‐poly‐L‐lysine production by multiple antibiotic‐resistance mutations and acidic pH shock optimization in Streptomyces albulus
Source: Microbiologyopen. 2018 Oct 8;8(5):e00728. doi: 10.1002/mbo3.728 (PMC6528598; doi:10.1002/mbo3.728)

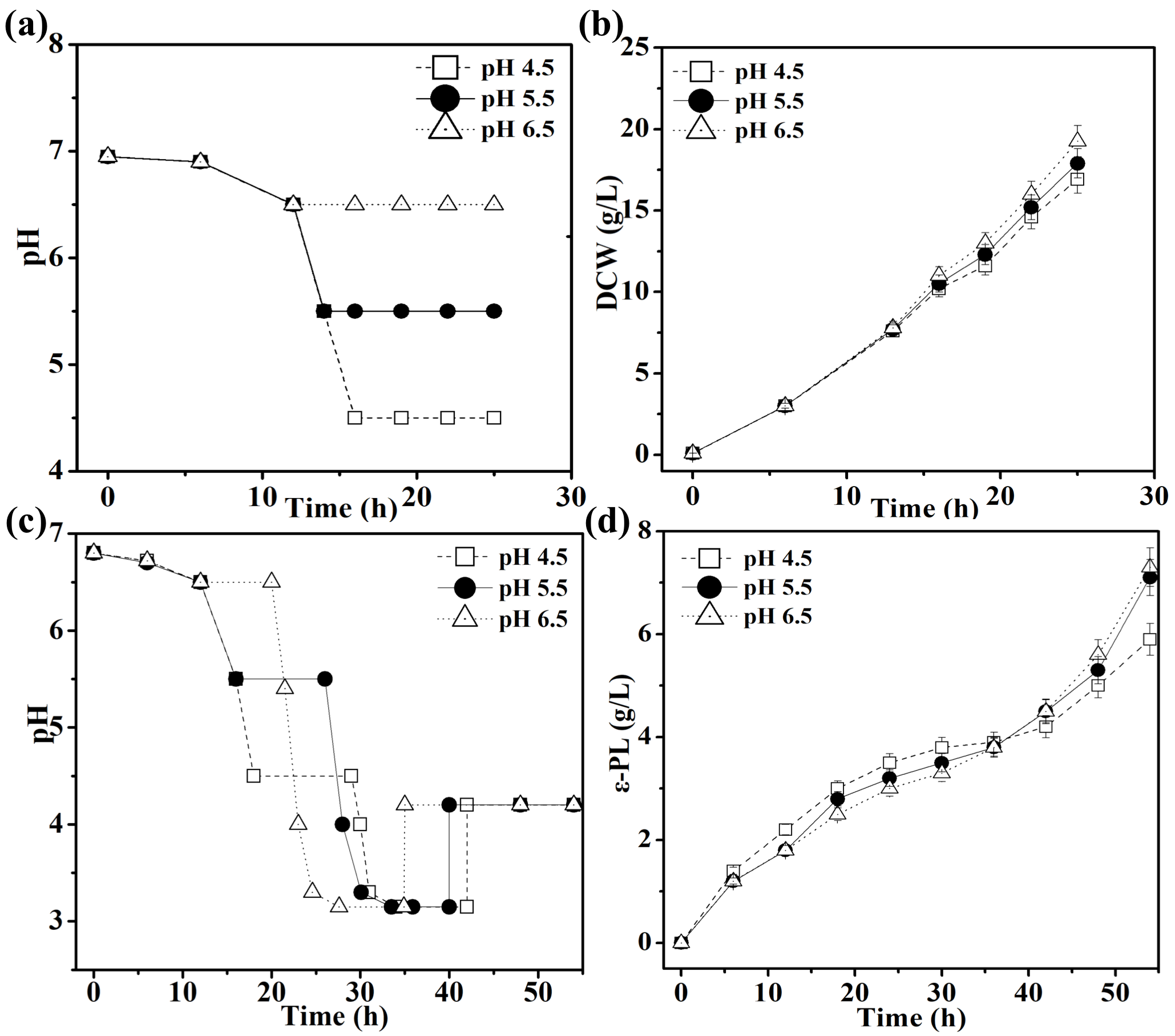

Supplement: Supplementary file 1 [file MBO3-8-e00728-s001.tiff]

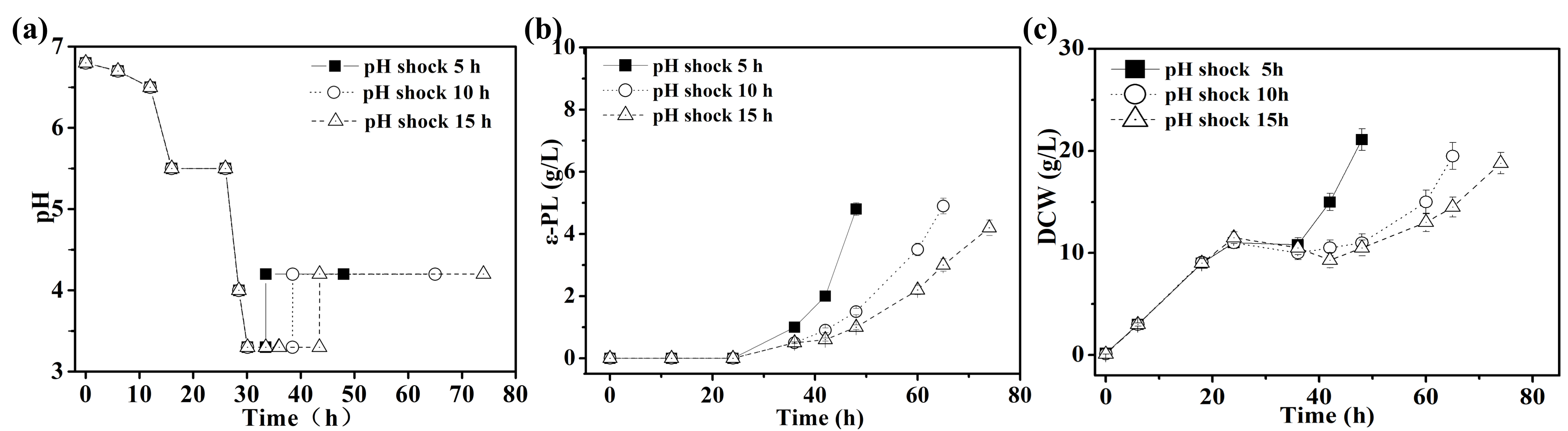

Supplement: Supplementary file 2 [file MBO3-8-e00728-s002.tiff]

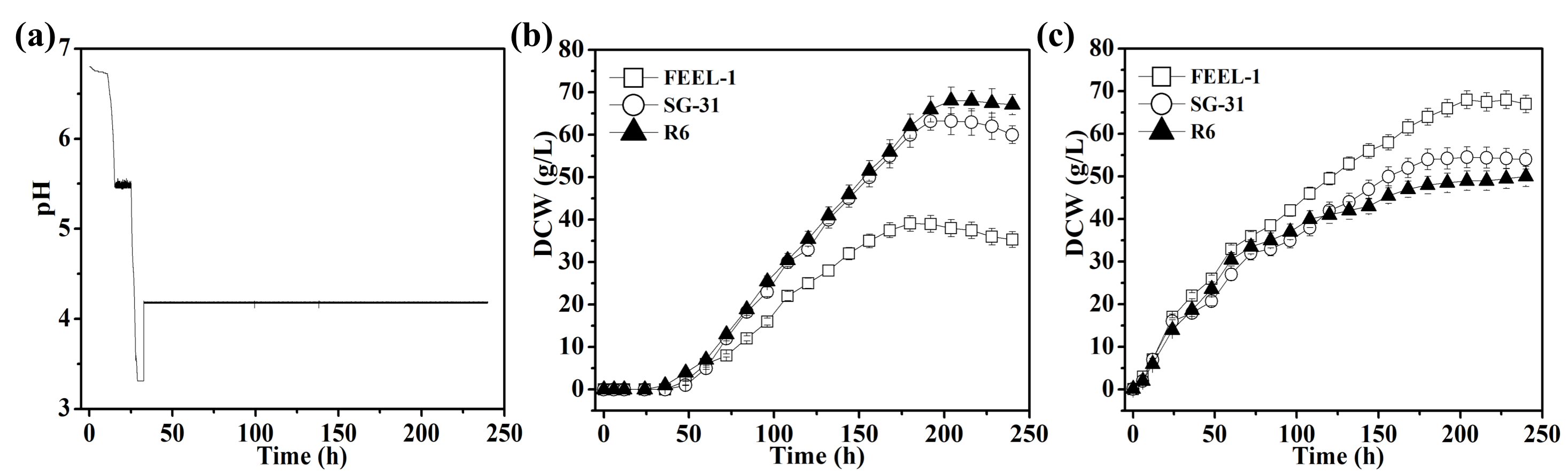

Supplement: Supplementary file 3 [file MBO3-8-e00728-s003.tiff]
