# Supplementary material for: Efficiently activated ε‐poly‐L‐lysine production by multiple antibiotic‐resistance mutations and acidic pH shock optimization in Streptomyces albulus
Source: Microbiologyopen. 2018 Oct 8;8(5):e00728. doi: 10.1002/mbo3.728 (PMC6528598; doi:10.1002/mbo3.728)
Supplement: Supplementary file 4 [file MBO3-8-e00728-s004.docx]

**APPENDIX_ TABLE S1**

**TABLE S1** ε-PL production and DCWs of the R6 mutant during sequential subculture

| Generation | DCW (g/L) | ε-PL production (g/L) |
| --- | --- | --- |
| 1 | 6.51±0.32 | 4.43±0.17 |
| 2 | 5.98±0.29 | 4.35±0.21 |
| 3 | 6.23±0.33 | 4.29±0.19 |
| 4 | 6.49±0.34 | 4.35±0.22 |
| 6 | 6.63±0.34 | 4.39±0.18 |
| 8 | 6.55±0.28 | 4.41±0.18 |

The fermentation was conducted in 24-well microtiter plates and the conditions were set as follows: inoculum size 8% (v/v), culture temperature of 30°C, initial pH pf 7.2 and YH medium volume of 2 mL. All assays were repeated three times.
