# Supplementary material for: Efficiently activated ε‐poly‐L‐lysine production by multiple antibiotic‐resistance mutations and acidic pH shock optimization in Streptomyces albulus
Source: Microbiologyopen. 2018 Oct 8;8(5):e00728. doi: 10.1002/mbo3.728 (PMC6528598; doi:10.1002/mbo3.728)
Supplement: Supplementary file 5 [file MBO3-8-e00728-s005.docx]

**APPENDIX FIGURE LEGENDS**

**FIGURE** **S1** Pre-acid-shock adaption at pH 6.5, 5.5 and 4.5. (a) and (b): Determination of the time consumption for different pre-acid-shock adaption modes. The pHs of culture broths were separately controlled at 6.5, 5.5 and 4.5, and the time when the DCW doubled was recorded. (c) and (d): Assessment of the optimal pH of pre-acid-shock adaption for ε-PL production. A 12 h pH shock was conducted after each pre-acid-shock adaption mode, ε-PL production of each mode was measured to choose the most appropriate pre-acid-shock adaption mode.

**FIGURE** **S2** Influence of pH shock intervals on the performance of R6 in batch fermentation. Different time intervals (5, 10 and 15 h) were maintained in a sequence of batch fermentations to determine the optimal pH shock interval for ε-PL synthesis. All assays were repeated three times and the error bars represent the standard deviation.

**FIGURE** **S3** Time course of the fermentation parameters of FEEL-1, SG-31 and R6 using the new acidic pH shock strategy. The assay was conducted under the following conditions: inoculum size of 8%, initial pH of 6.8.
